# Integrated Analysis of Long Non-coding RNAs (LncRNAs) and mRNA Expression Profiles Reveals the Potential Role of LncRNAs in Skeletal Muscle Development of the Chicken

**DOI:** 10.3389/fphys.2016.00687

**Published:** 2017-01-09

**Authors:** Zhenhui Li, Hongjia Ouyang, Ming Zheng, Bolin Cai, Peigong Han, Bahareldin A. Abdalla, Qinghua Nie, Xiquan Zhang

**Affiliations:** ^1^Department of Animal Genetics, Breeding and Reproduction, College of Animal Science, South China Agricultural UniversityGuangzhou, China; ^2^Guangdong Provincial Key Lab of Agro-Animal Genomics and Molecular Breeding and Key Lab of Chicken Genetics, Breeding and Reproduction, Ministry of AgricultureGuangzhou, China

**Keywords:** chicken, long non-coding RNA, *cis*-acting, *trans*-acting, skeletal muscle development

## Abstract

Long non-coding RNAs (lncRNAs) play important roles in transcriptional and post-transcriptional regulation. However, little is currently known about the mechanisms by which they regulate skeletal muscle development in the chicken. In this study, we used RNA sequencing to profile the leg muscle transcriptome (lncRNA and mRNA) at three stages of skeletal muscle development in the chicken: embryonic day 11 (E11), embryonic day 16 (E16), and 1 day after hatching (D1). In total, 129, 132, and 45 differentially expressed lncRNAs, and 1798, 3072, and 1211 differentially expressed mRNAs were identified in comparisons of E11 vs. E16, E11 vs. D1, and E16 vs. D1, respectively. Moreover, we identified the *cis*- and *trans*-regulatory target genes of differentially expressed lncRNAs, and constructed lncRNA-gene interaction networks. In total, 126 and 200 *cis*-targets, and two and three *trans*-targets were involved in lncRNA-gene interaction networks that were constructed based on the E11 vs. E16, and E11 vs. D1 comparisons, respectively. The comparison of the E16 vs. D1 lncRNA-gene network comprised 25 *cis*-targets. We determined that lncRNA target genes are potentially involved in cellular development, and cellular growth and proliferation using Ingenuity Pathway Analysis. The gene networks identified for the E11 vs. D1 comparison were involved in embryonic development, organismal development and tissue development. The present study provides an RNA sequencing based evaluation of lncRNA function during skeletal muscle development in the chicken. Comprehensive analysis facilitated the identification of lncRNAs and target genes that might contribute to the regulation of different stages of skeletal muscle development.

## Introduction

Embryonic patterning and organogenesis in metazoans entail complicated processes, including cellular differentiation, migration proliferation, and programmed cell death. These complex cellular and developmental processes depend on the precise spatiotemporal expression of regulatory factors. Evidence suggests that in complex organisms, RNA contains a hidden layer of regulatory information. It not only functions as a messenger between DNA and the protein, but also plays a role in the regulation of genome organization and gene expression (Morris and Mattick, 2014). Data from high-throughput sequencing studies have revealed that only a very small percentage (1–2%) of the mammalian genome encodes proteins, but tens of thousands of intergenic sites are transcribed to non-coding RNA. This transcription plays a critical role in regulating gene expression during several biological processes (Muers, 2011). Many small non-coding RNAs, such as microRNAs (miRNAs), PIWI-interacting RNAs (piRNAs), and small nucleolar RNAs (snoRNAs) have been the subject of many functional studies; however, systematic identification of the functions of lncRNA has yet to be conducted.

LncRNAs, or mRNA-like long non-coding RNAs, are a novel class of regulatory RNAs, with sizes ranging from 200 bp to >100 kb (Novikova et al., 2013). LncRNAs are transcribed by RNA polymerase II, but do not encode proteins. There is evidence to suggest that lncRNAs, by a variety of mechanisms, serve as versatile regulators of diverse aspects of biology. The *Xist* gene is a master regulatory switch that controls the inactivation of the X chromosome in mammals, and is one of the first known examples of a lncRNA that directly participates in the formation of repressive chromatin (Lee and Bartolomei, 2013). Recently, specific roles of lncRNAs in the development of diverse organs and tissue types have been identified. For example, Bvht and Fendrr are two important lncRNAs that are involved in cardiac development in the mouse. In neonatal cardiomyocytes, knockdown of Bvht by RNAi changes cardiac-specific gene expression and inhibits normal development to mature cardiomyocytes (Klattenhoff et al., 2013). Similarly, knockout of Fendrr impairs heart function, leads to deficits in the body wall, and ultimately results in embryonic lethality (Grote et al., 2013). In addition to their extensive roles in organ development, lncRNAs are also known to play a role in skeletal muscle differentiation. Linc-MD1 is one of the first lncRNAs that was observed to be involved in myogenesis. This lncRNA acts as a competing endogenous RNA and “sponges” miR-133 and miR-135 to regulate the expression of MAML1 and MEF2C, which are transcription factors that activate late-differentiation muscle genes (Cesana et al., 2011). Moreover, lncRNA H19, which is highly expressed in the developing embryo and in adult muscle, functions as a molecular sponge for the let-7 family of miRNAs, and thereby regulates muscle differentiation (Kallen et al., 2013). LncRNAs can be *cis*-acting or *trans*-acting, and the location of the target gene is commonly used to distinguish between the two. *Cis*-acting lncRNAs regulate the expression of target genes that are located at or near the same genomic locus, whereas *trans*-acting lncRNAs can either inhibit or activate gene transcription at independent chromosomal loci (Fatica and Bozzoni, 2014). For example, lincRNA-p21 activates *p21* expression in *cis* to influence the chromatin state, activate *p53* pathway genes, and enforce the G1/S checkpoint (Dimitrova et al., 2014). In addition, the lncRNA Six3OS acts in *trans* to regulate retinal development by modulating *Six3* activity (Rapicavoli et al., 2011).

Skeletal muscle development in the chicken is a multi-step process that comprises four major stages: in the initial stage, muscle precursor cells differentiate from the somite; in the second stage, myogenic precursor proliferation, and differentiation give rise to myoblasts; in the third stage, myoblast differentiation gives rise to myotubes; and finally, myofibers are formed from the myotubes (Luo et al., 2013). Many genes, such as paired box 3 (*Pax3*), paired box 7 (*Pax7*), myogenic differentiation (*MyoD*), myogenic factor 5 (*Myf5*), and myogenin (*MyoG*) (Sobolewska et al., 2011) are involved in the regulation of skeletal muscle development in the chicken; however, research on the role of lncRNAs in the regulation of this process remains scarce. With the aid of non-coding RNA (ncRNA) library construction, 125 ncRNAs have been identified in the chicken that play important roles in tissue development and lineage/species specification during evolution (Zhang et al., 2009). In another study, 281 new intergenic lncRNAs were identified in chicken embryo skeletal muscle, and further analyses suggested that these lncRNAs were less conserved than protein-coding genes, but slightly more conserved than random non-coding sequences (Li T. et al., 2012). Although an increasing number of lncRNAs are being characterized by high-throughput sequencing studies, few examples of regulatory mechanisms involving lncRNAs in chicken muscle development have been described.

In this study, we identified lncRNA and mRNA expression profiles in three different stages of chicken skeletal muscle development. Differentially expressed lncRNAs were then used in bioinformatics analyses to predict *cis*- and *trans*-target genes. These findings were integrated with differentially expressed mRNA data to improve the accuracy of target prediction. The predicted *cis*- and *trans*-targets were then used for the construction of lncRNA-gene correlation networks. Ingenuity Pathway Analysis (IPA) and gene ontology (GO) analysis were performed to investigate the functions associated with network genes. In summary, this study provides predictions about the functional interactions of lncRNAs and protein-coding genes, and the information generated from these predictions can be utilized in further studies of lncRNA function in chicken skeletal muscle development.

## Materials and methods

### Ethics statement

All animal experiments were performed according to protocols approved by the Institutional Animal Care and Use Committee at the South China Agricultural University (Guangzhou, People's Republic of China). All experiments were conducted in compliance with the regulations and guidelines established by that committee.

### Chicken embryo incubation and tissue collection

Fertilized eggs of a native Chinese yellow meat-type chicken known as Xinghua chickens were used in the this study. The Xinghua chicken is an indigenous chicken breed from Xinghua town, Fengkai County, Guangdong province, China is characterized by a relatively slow growth rate. The fertilized eggs were incubated in a humidified incubator at 37.5°C and 65% humidity. Skeletal leg muscles were resected at embryonic day 11 (E11), embryonic day 16 (E16), and 1 day after hatching (D1). All tissues were immediately frozen in liquid nitrogen and stored at −80°C until RNA extraction. The sex of the chicken was determined by PCR amplification of the *CHD1* gene using sex-specific primers (Li Z. L. et al., 2012). Chickens with two bands of 600 bp and 450 bp were born as females, whereas those with one band of 600 bp were born as males. The sex-specific primer sequences are presented in Table 1.

**Table 1:** **Sex-specific primers and real-time qPCR primers**.

**Gene and lncRNA**		**Primer sequence (5′−3′)**	**Product length (bp)**	**Annealing temperature (°C)**
Sex-specific primers	CHD1-F	GTTACTGATTCGTCTACGAGA	600 or 450	55
	CHD1-R	ATTGAAATGATCCAGTGCTTG		
qPCR primers	TEAD4-F	CTTTCCTTCTTCTCACCCTC	168	53
	TEAD4-R	TAGCAACCCTTCGTCCTT		
	DMD-F	ATGATTTTCGAGATGGACG	81	62
	DMD-R	TGGAGCCCTTTTCTTTTG		
	FGF13-F	GCTATACAGCAGGCAAGG	118	62
	FGF13-R	CACTCGTAAACCCACAGG		
	DLK1-F	CAGGAAAGGACCATGTATTA	100	52
	DLK1-R	AGGGCATAGACAGGAAGC		
	lnc00003323-F	ACATCTTTATCCGTGCTG	197	57
	lnc00003323-R	AAGACTTACTCCAGGTGGT		
	lnc00005738-F	CACCTGTAATAAACCCATAA	185	57
	lnc00005738-R	CTCCCACTTTAGTAAACTCC		
	lnc00023378-F	TTCCATGAACAGCACCCA	128	57
	lnc00023378-R	AGATGAGCAGCGATAACACC		
	lnc00073545-F	CTTCTAGCAAGCCTAATG	118	52
	lnc00073545-R	CTAACAAACCCTAACTCCT		

### Illumina deep sequencing and sequence analysis

Total RNA for RNA sequencing (RNA-seq) was isolated from six chicken leg muscle samples (two each from E11, E16, and D1), using Trizol Reagent (Invitrogen, Carlsbad, CA, USA) according to the manufacturer's protocol. RNA quantity and quality were evaluated on an Agilent 2100 Bioanalyzer (Agilent Technologies, Waldbronn, Germany), and RNA integrity was further examined using agarose gel electrophoresis. Ribosomal RNA (rRNA) was removed from the total RNA using the Epicentre Ribo-Zero™ rRNA Removal Kit (Epicentre, Madison, Wisconsin, USA) following the manufacturer's instructions. High-throughput RNA-seq was performed on the Illumina Hiseq 2500 platform (Illumina, San Diego, CA, USA). The Illumina sequencing raw reads were cleaned by removing empty reads, adapter sequences, reads with over 10% N sequences, and low quality reads, in which the number of bases with a quality value *Q* ≤ 10 was >50%. In addition, rRNA reads were identified, by blasting against the rRNA database (http://www.arb-silva.de/) using Simple Object Access Protocol (SOAP) software, and removed from the dataset. The filtered reads were mapped to the chicken reference genome (ftp://ftp.ensembl.org/pub/release-73/fasta/gallus_gallus/dna/) using the Tophat2 software. The mapped reads were assembled and transcripts were constructed using the Cufflinks 2.0.2 software. The Ensembl, NCBI RefGene, and UCSC databases were chosen as annotation references for lncRNA analyses. The Coding Potential Calculator (CPC), Coding-Non-Coding Index (CNCI) and Phylogenetic codon substitution frequency (PhyloCSF) were applied to estimate coding ability of potential lncRNAs. An RNA length ≥ 200 nt, CPC score ≤ 0, CNCI score ≤ 0, and PhyloCSF score ≤ −20 were used to evaluate the coding potential of transcripts. The RefSeq and Ensembl databases were used for gene analysis. The expression levels of the transcripts were expressed as FPKM (fragments per kilobase of transcript per million mapped reads) values using the Cuffnorm program. Differentially expressed lncRNAs and mRNAs for each comparison (E11 vs. E16, E11 vs. D1, and E16 vs. D1) were identified using Cuffdiff (the differential expression analysis tool packaged with the Cufflinks software), with a *q* < 0.05 and |(fold change)| ≥ 2 as the cut-off points. The sequencing data obtained from RNA-seq were released to the National Center for Biotechnology Information (NCBI) Gene Expression Omnibus (GEO) database under the accession number GSE91060.

### *Cis*- and *trans*-analyses

Differentially expressed lncRNAs were selected for *cis*- and *trans*-target gene prediction, and these findings were integrated with differentially expressed mRNA data to improve the accuracy of target prediction. To classify lncRNA *cis*-target genes, we used the UCSC Genome Bioinformatics tool to identify differentially expressed genes (DEGs) located ~300 kb upstream and downstream of differentially expressed lncRNAs, and determined the potential that the lncRNA was *cis*-acting. To classify lncRNA *trans*-target genes, the BLAST (*e* < 1E − 5) software was used to assess the impact of lncRNA binding on complete mRNA molecules. The RNAplex program (−e −20) was then used to identify possible *trans*-target genes of the lncRNAs. Differentially expressed lncRNAs and their corresponding differentially expressed *cis*- and *trans*-target genes were used to construct lncRNA-gene interaction networks using the Cytoscape program.

### IPA, gene ontology, and pathway analysis

Gene interaction network and biological process enrichment analyses for the *cis*- and *trans*-target genes of differentially expressed lncRNAs were performed using IPA (www.ingenuity.com). Ingenuity Pathway Analysis is a web-based software from Ingenuity Systems® (Qiagen, Redwood City, CA, USA) that enables investigators to analyze data derived from high-throughput sequencing. IPA provides a comprehensive database of known networks and pathways that are continuously being updated based on published work on gene functions and interactions. Each gene identifier was imported and mapped to its corresponding gene object in the Ingenuity Pathways Knowledge Base (IPKB) and superimposed onto a global molecular network, based on the information stored in the IPKB. IPA uses computational algorithms to identify local networks that are particularly enriched for the genes of interest. A network score that is used to rank networks is also provided. This numerical score, which is based on the hyper-geometric distribution, is calculated using a right-tailed Fisher's exact test and is represented as the negative log of the *P*-value. Functional enrichment analysis was also performed on these networks to understand the significance of the biological functions and/or disease phenotypes of the genes in the network. The predicted lncRNA target genes were entered into the Molecule Annotation System (MAS) 3.0 (http://bioinfo.capitalbio.com/mas3), which is based on the Kyoto Encyclopedia of Genes and Genomes database (KEGG) (CapitalBio, Beijing, China). The MAS uses GO data as an advantage to identify the molecular functions represented in the gene profile. The MAS 3.0 software was used to perform GO and KEGG analyses.

### Real-time qPCR analysis

Single-strand cDNA was synthesized from 1 μg of total RNA using the PrimeScript™ RT reagent kit with gDNA Eraser (Takara, Osaka, Japan). The qPCR reactions were performed on a Bio-Rad CFX96 Real-Time Detection system to detect the RNA expression level of each gene, using the iTaq™ Universal SYBR® Green Supermix kit (Bio-Rad Laboratories Inc., Hercules, CA, USA), for which the chicken β*-actin* gene was used as an internal control. Data analysis was carried out with the 2^−ΔΔCt^ method as described previously (Livak and Schmittgen, 2001). Real-time qPCR primers were designed using the Premier Primer 5.0 software (Premier Biosoft International, Palo Alto, CA, USA). The qPCR primer sequences are presented in Table 1.

### Statistical analysis

Quantitative expressions of the data are presented as mean ± S.E.M of three independent experiments. In each experiment, the assay was performed in triplicate. Statistical significance of the data was tested by performing one-sample or paired *t*-tests when required. When applicable, the specific types of tests and the *P* < 0.05 are indicated in the figure legends.

## Results

### Overview of RNA-sequencing

The Illumina Hiseq 2500 platform was used to perform RNA-seq for the six cDNA libraries, and 125 bp paired-end reads were generated. The number of raw reads generated from each library was exceeded 100 million reads (Table 2). After filtering the low quality reads, most of the clean reads still comprised more than 90% of the raw data, with the exception of the E16-2 group (85.13%) and D1-1 group (89.47%). More than 73.70% of the clean reads were perfectly mapped to the chicken reference genome. The unique mapped reads ranged from 95.62 to 96.54% of the total mapped reads (Table 2).

**Table 2:** **Summary of draft reads of six libraries by RNA-sequencing**.

**Sample**	**Raw reads**	**Clean reads**	**Total residues (bp)**	**Total mapped reads**	**Unique mapped reads**
E11-1	120,024,130	109,367,542 (91.12%)	14,797,215,325	81,510,188 (74.50%)	78,561,396 (96.38%)
E11-2	108,620,754	99,583,044 (91.68%)	13,488,389,034	73,440,167 (73.70%)	70,902,173 (96.54%)
E16-1	104,403,766	98,171,720 (94.03%)	13,415,537,569	74,087,627 (75.50%)	70,893,952 (95.68%)
E16-2	123,268,886	104,948,578 (85.13%)	14,065,328,305	78,594,049 (74.90%)	75,172,271(95.65%)
D1-1	127,403,762	113,984,606 (89.47%)	15,479,331,668	84,119,306 (73.80%)	80,432,724 (95.62%)
D1-2	123,022,016	117,366,974 (95.40%)	16,020,192,921	88,208,260 (75.20%)	84,401,995 (95.68%)

### Identification of lncRNAs in chicken leg skeletal muscle

**Figure 1:**
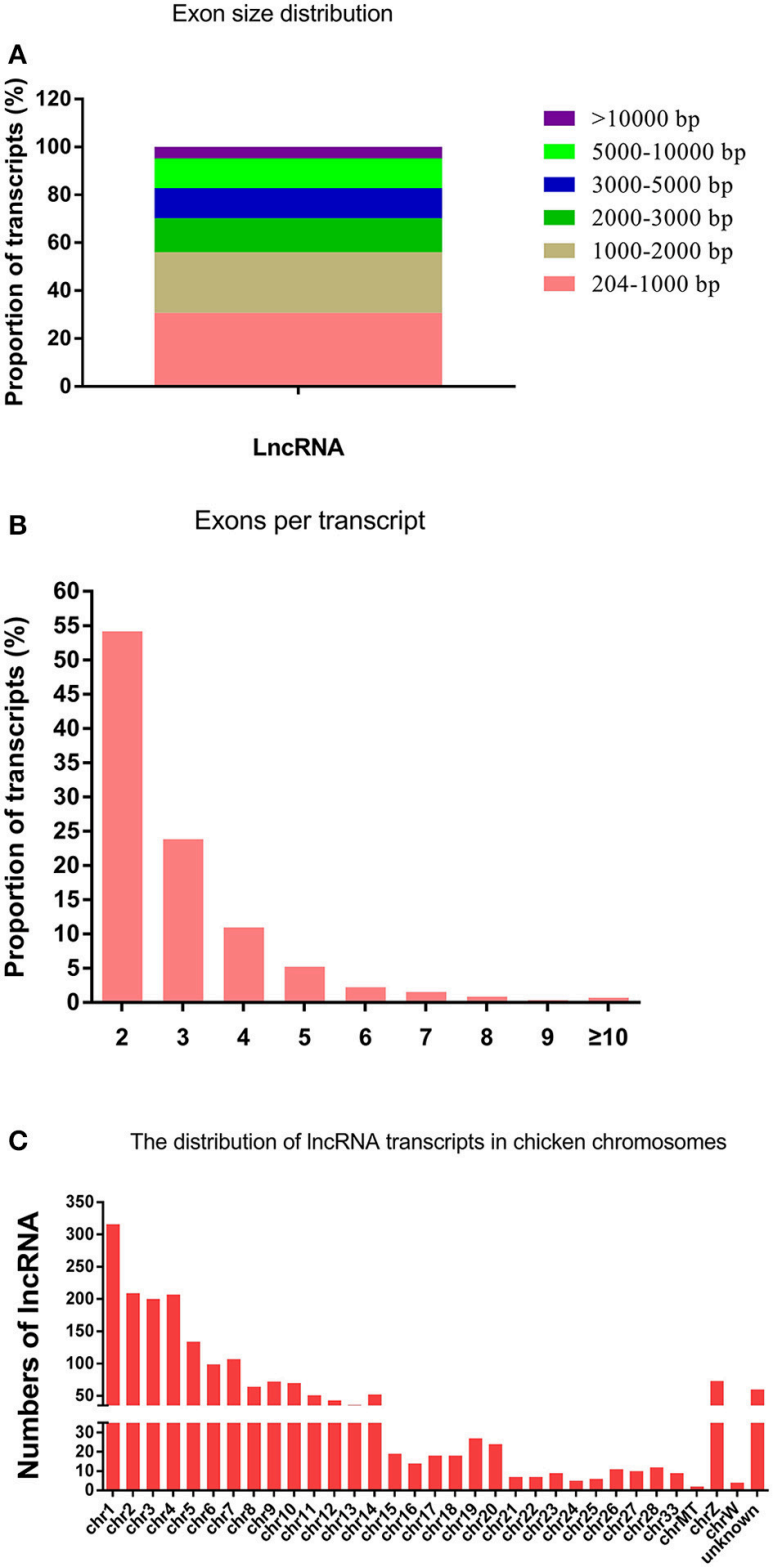
**The features of chicken lncRNAs. (A)** Exon length distribution of chicken lncRNAs. **(B)** Exon numbers per transcript of chicken lncRNAs. **(C)** Chromosome distribution of lncRNA transcripts identified in chicken leg muscle.

After a stringent filtering process to discard transcripts that did not have the characteristics of lncRNAs, RNA-seq yielded 1995 lncRNA transcripts. The length of the lncRNAs ranged from 204 to 32984 bp, with 30.78 and 25.31% of lncRNAs having a length of 204–1000 and 1000–2000 bp, respectively (Figure 1A). The lncRNA genes had an average length of 2941 bp and 2.9 exons (Figures 1A,B). The 1995 lncRNA transcripts were distributed among chicken chromosomes 1–28, 33, MT, Z, and W. Quite notably, 316 lncRNAs were located in the chromosome 1 (Figure 1C).

### Differential expression analysis of lncRNAs and mRNAs

**Figure 2:**
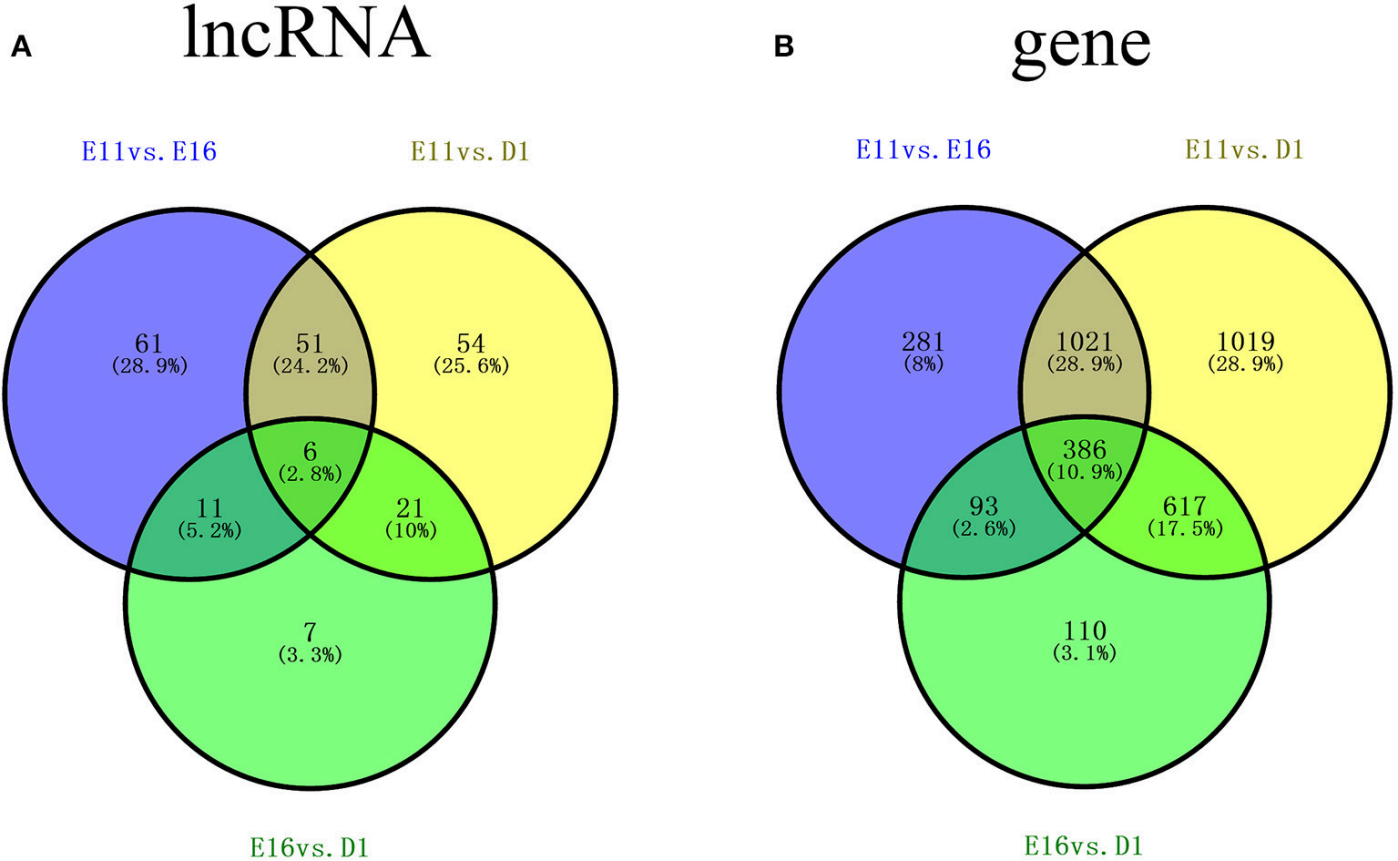
**Venn diagrams of differentially expressed lncRNAs (DE-lncRNAs) and differentially expressed genes (DEGs) showing comparisons of three stages of skeletal muscle development in the chicken (A)** Number of DE-lncRNAs for three comparisons: E11 vs. E16, E11 vs. D1, and E16 vs. D1. **(B)** Numbers of DEGs for three comparisons: E11 vs. E16, E11 vs. D1, and E16 vs. D1. E11, E16, and D1 indicate the developmental stages of skeletal muscle development: embryonic day 11, embryonic day 16, and 1 day after hatching, respectively.

To investigate the key lncRNAs and mRNAs involved in chicken skeletal muscle development, RNA-seq (with rRNA removed) was performed to detect the differentially expressed lncRNAs (DE-lncRNAs) and DEGs at three stages of leg muscle development in the chicken (E11, E16, and D1). DE-lncRNAs and DEGs were identified by Cuffdiff analysis (*q* < 0.05; |(fold change)| ≥ 2). Among three comparisons, E11 vs. E16, E11 vs. D1, and E16 vs. D1, 129, 132, and 45 DE-lncRNAs were detected, respectively. Among these DE-lncRNAs, 26, 24, and 6 lncRNAs were uniquely expressed in one of the two samples in the E11 vs. E16 (Table S1), E11 vs. D1 (Table S2), and E16 vs. D1 (Table S3) comparisons, respectively. Of these DE-lncRNAs, six, including lnc00060568, lnc00057952, lnc00019955, lnc00064868, lnc00002354, and lnc00029488, were identified as differentially expressed among all three comparisons, whereas 61, 54, and 7 lncRNAs were found exclusively in the E11 vs. E16, E11 vs. D1, and E16 vs. D1 comparisons, respectively (Figure 2A).

Comparisons of gene expression levels among the three developmental stages revealed that there were 1798 DEGs (*q* < 0.05; |(fold change)| ≥ 2) between E11 and E16 (E11 vs. E16), 3072 DEGs between E11 and D1 (E11 vs. D1), and 1211 DEGs between E16 and D1 (E16 vs. D1). Among these DEGs, 27, 50, and 5 genes were uniquely expressed in one of the two samples in the E11 vs. E16 (Table S4), E11 vs. D1 (Table S5), and E16 vs. D1 (Table S6) comparisons, respectively. Of these DEGs, 386 genes were detected in all three comparisons, whereas 281, 1019, and 110 genes were exclusively differentially expressed in E11 vs. E16, E11 vs. D1, and E16 vs. D1 comparisons, respectively (Figure 2B).

### lncRNA-gene interaction network construction

**Figure 3:**
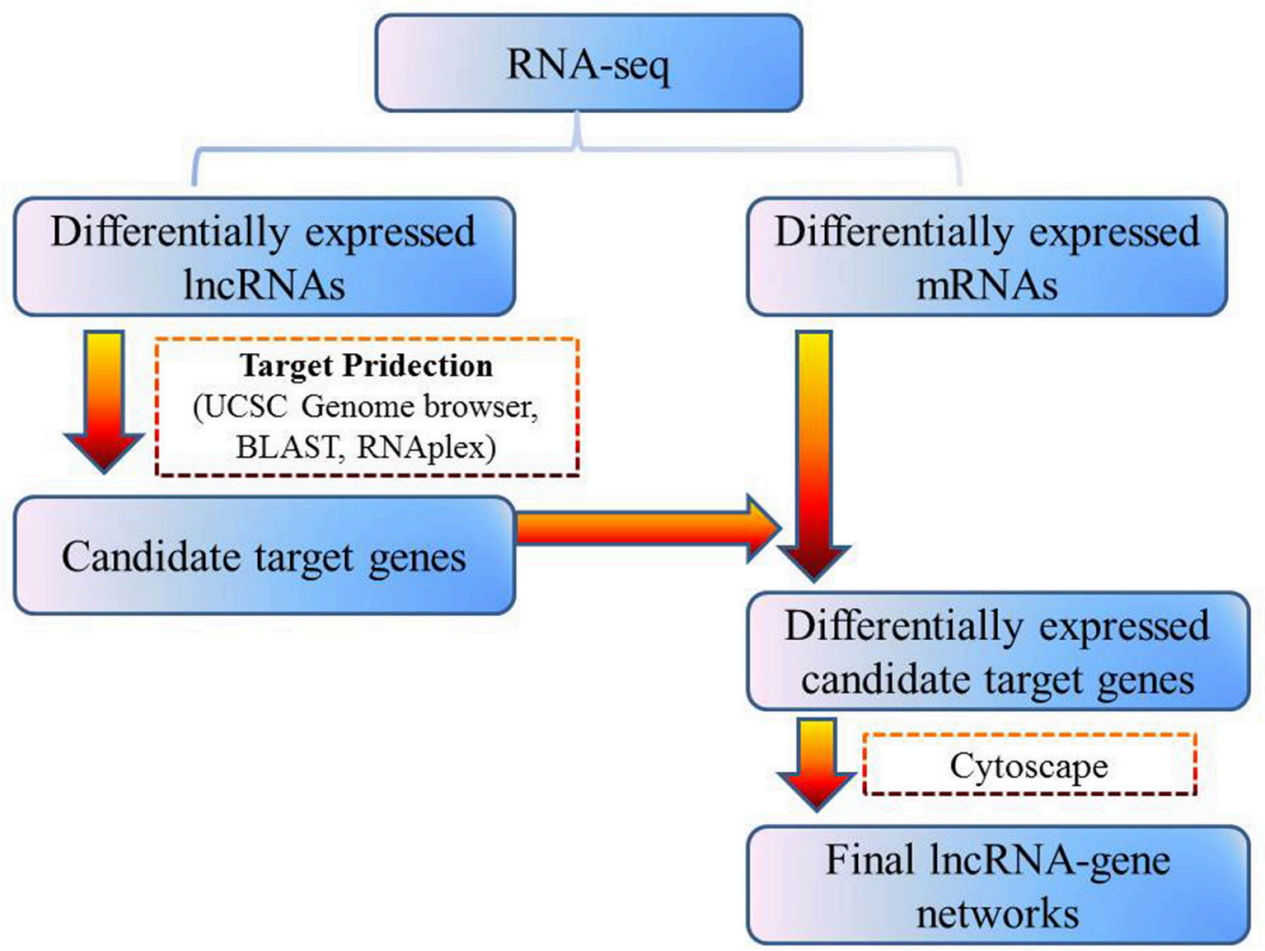
**Schematic of lncRNA-gene interaction network construction**.

**Figure 4:**
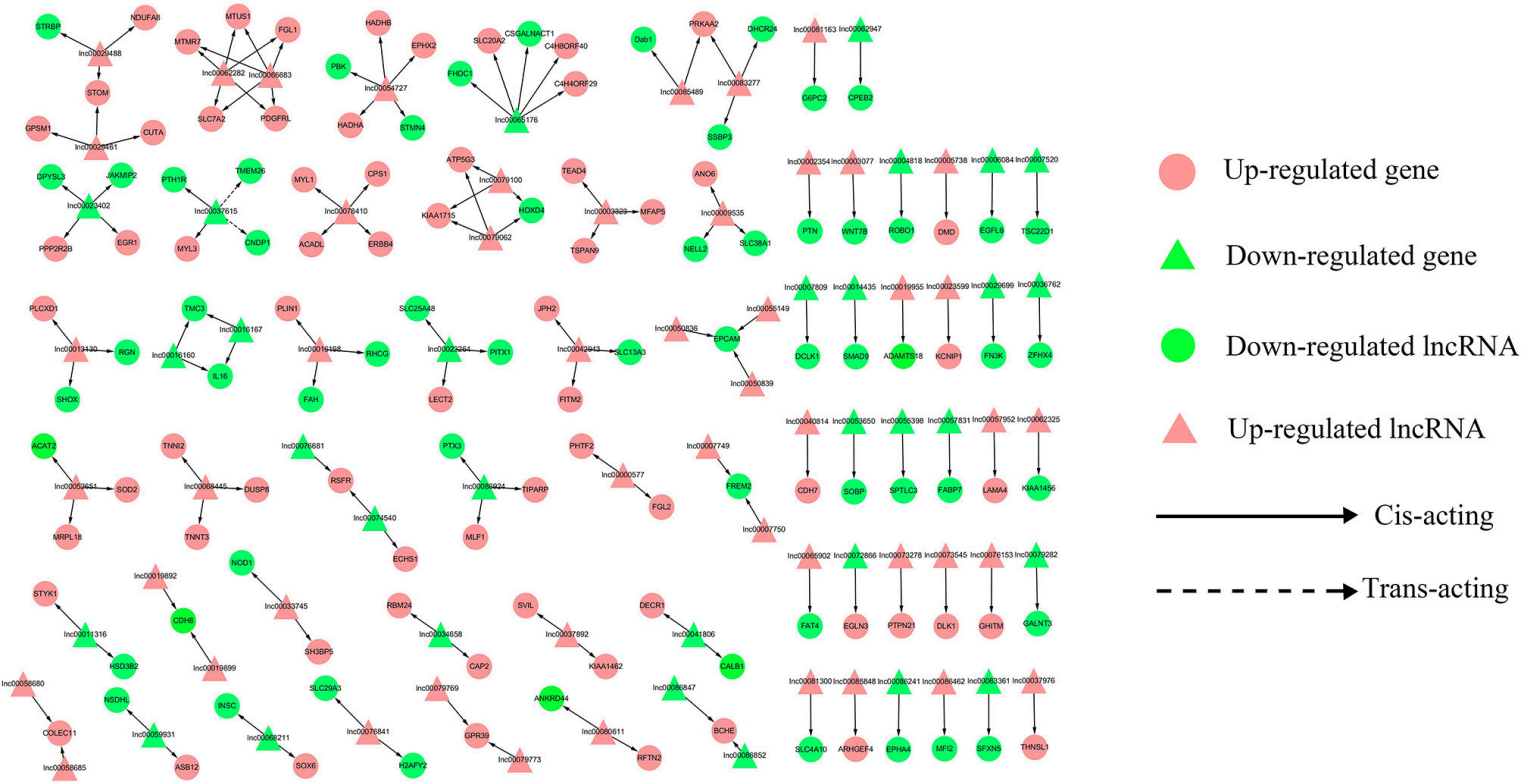
**LncRNA-gene network for comparison of E11 vs. E16**. E11 vs. E16, differentially expressed lncRNAs and their corresponding differentially expressed *cis*- and *trans*-target genes were used to construct a lncRNA-gene interaction network. In this network, up-regulated genes are displayed as pink circles, down-regulated genes are displayed as green circles, up-regulated lncRNAs are displayed as pink triangles, and down-regulated lncRNAs are displayed as green triangles. Solid lines represent the interactions between differentially expressed lncRNAs and their corresponding *cis* target genes, whereas the dashed lines represent interactions between differentially expressed lncRNAs and their corresponding *trans*-target genes.

**Figure 5:**
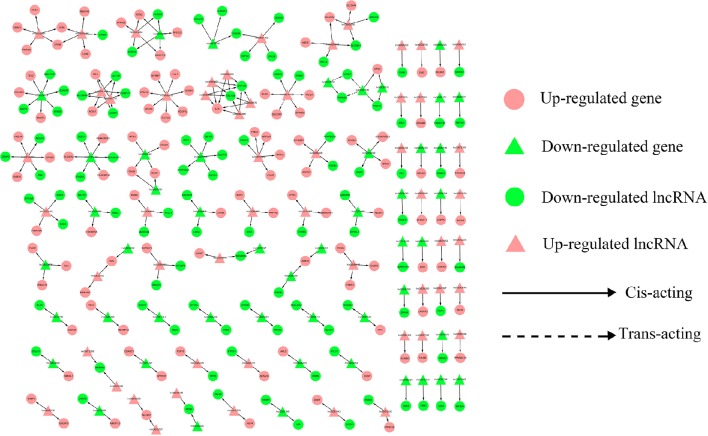
**LncRNA-gene network for comparison of E11 vs. D1**. E11 vs. D1, differentially expressed lncRNAs and their corresponding differentially expressed *cis*- and *trans*-target genes were used to construct a lncRNA-gene interaction network. In this network, up-regulated genes are displayed as pink circles, down-regulated genes are displayed as green circles, up-regulated lncRNAs are displayed as pink triangles, and down-regulated lncRNAs are displayed as green triangles. Solid and dashed lines represent the interactions between differentially expressed lncRNAs and their corresponding *cis*- and *trans*-target genes, respectively.

**Figure 6:**
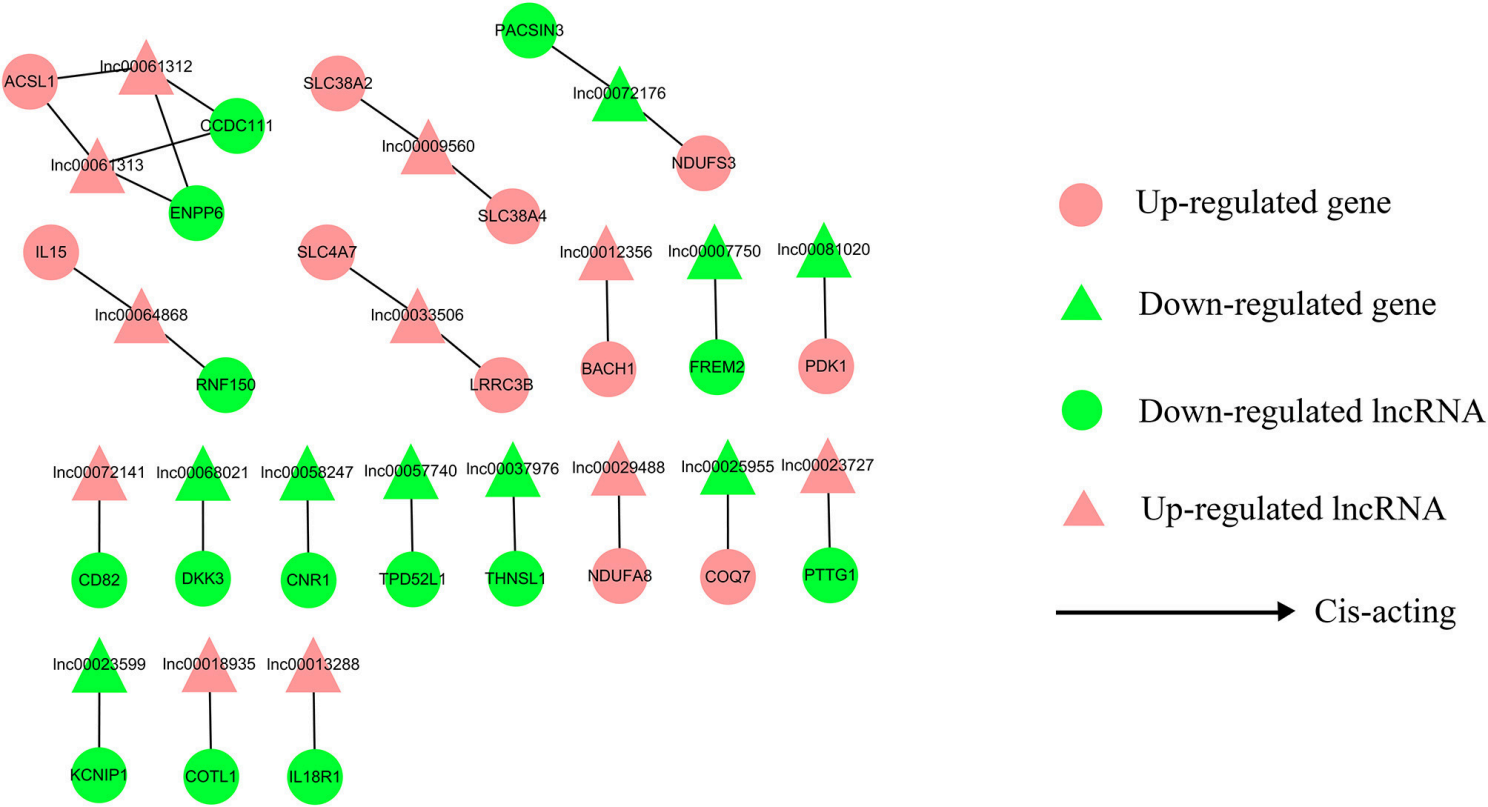
**LncRNA-gene network for comparison of E16 vs. D1**. E16 vs. D1, differentially expressed lncRNAs and their corresponding differentially expressed *cis*-target genes were used to construct a lncRNA-gene interaction network. In this network, up-regulated genes are displayed as pink circles, down-regulated genes are displayed as green circles, up-regulated lncRNAs are displayed as pink triangles, and down-regulated lncRNAs are displayed as green triangles. Solid lines represent the interactions between the differentially expressed lncRNAs and their corresponding *cis*-target genes.

To address the question of how lncRNAs function in concert with their target genes (mRNAs) to regulate chicken muscle development, and to identify the key molecular players in this process, *cis*- and *trans*-targets of the differentially expressed lncRNAs were predicted, and the possible regulatory networks of interactions between lncRNAs and their target genes (mRNAs) were constructed. Previous studies have confirmed that lncRNAs regulate the expression of neighboring protein-coding genes through *cis*-acting mechanisms (Bu et al., 2012; Han et al., 2012). In addition, lncRNAs can regulate the expression of genes that are located on other chromosomes; such regulation is called *trans*-acting (Han et al., 2012). In the present study, bioinformatics analysis was used to predict potential *cis*- and *trans*-target genes of DE-lncRNAs, and further construct a lncRNA-gene interaction network between the DE-lncRNAs and their corresponding differentially expressed *cis*- and *trans*-target genes. Construction of the lncRNA-gene network consisted of three steps (Figure 3). Firstly, the genes within 300 kb upstream and downstream of the lncRNA were identified using the UCSC Genome Browser. Among these genes, those that were coexpressed with the lncRNA were classified as potential lncRNA *cis*-target genes. Secondly, BLAST and RNAplex software were used to identify *trans*-target genes. To narrow the field and improve the accuracy of target prediction, only those putative *trans*-target genes that were coexpressed with the lncRNAs were classified as *trans*-lncRNA target genes. Thirdly, Cytoscape was used to construct a lncRNA-gene interaction network based on the DE-lncRNAs and their corresponding differentially expressed *cis*- and *trans*-target genes. For the E11 vs. E16 comparison, the lncRNA-gene interaction network comprised 211 network nodes and 148 lncRNA-gene connections among 83 lncRNAs and 128 protein-coding genes (Figure 4, Table S7). For the E11 vs. D1 comparison, the lncRNA-gene interaction network comprised 305 network nodes and 242 lncRNA-gene connections among 102 lncRNAs and 203 protein-coding genes (Figure 5, Table S8). For the E16 vs. D1 comparison, the lncRNA-gene interaction network comprised 46 network nodes and 28 lncRNA-gene connections among 21 lncRNAs and 25 protein-coding genes (Figure 6, Table S9).

We then analyzed the expression correlation between the network lncRNAs and their corresponding target genes. In the network constructed from DE-lncRNAs and target genes identified from the E11 vs. E16 comparison, 92 lncRNA-gene connections were positively correlated, whereas another 56 connections were negatively correlated (Figure 4, Table S7). For the E11 vs. D1 comparison, 138 lncRNA-gene connections were positively correlated and 104 connections were negatively correlated (Figure 5, Table S8). For the E16 vs. D1 comparison, 16 lncRNA-gene connections were positively correlated and 12 connections were negatively correlated (Figure 6, Table S9). Directionality analysis showed that for all three contrast networks, the number of positive correlations between lncRNA-gene pairs was higher than the number of negative correlations.

### Ingenuity pathway analysis

**Figure 7:**
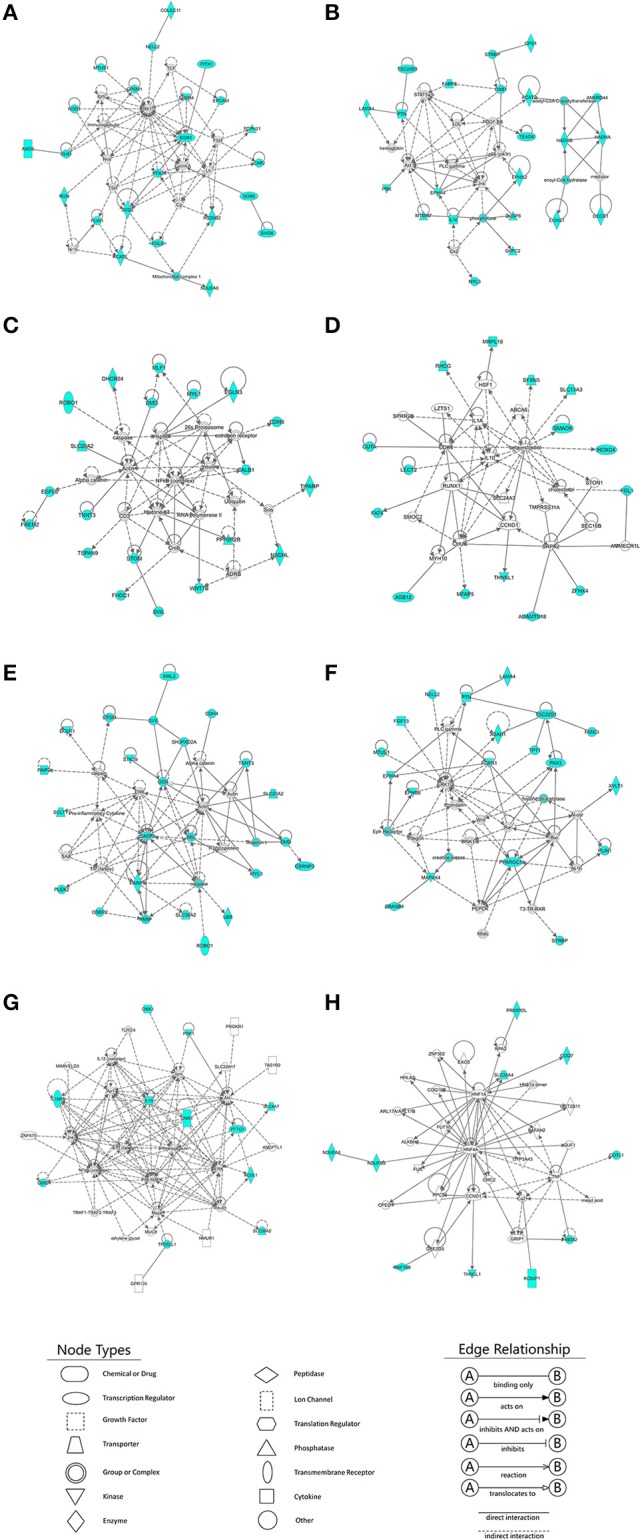
**Ingenuity Pathway Analysis (IPA) identification of gene networks affected by differentially expressed lncRNA (DE-lncRNA) target genes**. IPA online software was used to identify the gene-gene interaction networks, using differentially expressed DE-lncRNA target genes as input. **(A–D)** Four major gene networks identified for the E11 vs. E16 comparison. **(E,F)** Two major gene networks identified for the E11 vs. D1 comparison. **(G,H)** Two major gene networks identified for the E16 vs. D1 comparison. Nodes shaded in blue represent the DE-lncRNA target genes that are also involved in the IPA interaction network. White nodes represent transcription factors (as opposed to DE-lncRNAs target genes) that are associated with the regulation of some of these genes, based on the evidence stored in the IPA knowledgebase. Edges and nodes are annotated with labels that illustrate the nature of the relationship between the genes and their functions.

In total, 128, 203, and 25 DEGs that were also targets of DE-lncRNAs belonged to the E11 vs. E16, E11 vs. D1, and E16 vs. D1 comparison lncRNA-gene networks, respectively. To identify the functions of the DE-lncRNAs target genes and their potential network connections, we used IPA to determine which gene networks might be affected by these DE-lncRNAs target genes. For the E11 vs. E16 comparison, we identified a total of ten networks, and the top four were related to cancer, organismal injury and abnormalities, cell cycle, developmental disorders, hereditary disorders, metabolic disease, reproductive system disease, cellular development, cellular growth and proliferation, and connective tissue development and function (Figures 7A–D, Table 1). In total, 23 (Figure 7A), 22 (Figure 7B), 20 (Figure 7C), and 15 (Figure 7D) DEGs belonged to these top four gene-gene networks. For the E11 vs. D1 comparison, 16 networks were identified and the top two were related to cell morphology, organ morphology, reproductive system development and function, embryonic development, organismal development, and tissue development (Figures 7E,F, Table 1). A total of 22 (Figure 7E), and 19 (Figure 7F) DEGs belonged to these top two gene-gene networks. In addition, for the E16 vs. D1 comparison, we identified a total of four networks, and the top two were related to nervous system development and function, cellular development, cellular growth and proliferation, developmental disorders, hereditary disorders, and ophthalmic disease (Figures 7G,H, Table 1). A total of 11 (Figure 7G), and 10 (Figure 7H) DEGs belonged to these top two gene-gene networks.

### GO and KEGG pathway analysis

We then conducted GO and KEGG pathway analyses to gain insight into the functions of DE-lncRNA target genes. GO analysis was done to uncover enriched (*q* < 0.01) biological process terms for each contrast. A total of 107 enriched GO terms, including 43 for E11 vs. E16 (Figure S1A, Table S10); 51 for E11 vs. D1 (Figure S1B, Table S11); and 13 for E16 vs. D1 (Figure S1C, Table S12), were identified. Among these terms, 71 were unique GO terms that each appeared only once among all three comparisons. Many of the GO terms that were enriched in more than one comparison were related to muscle maintenance, lysine transport, ornithine transport, arginine transport, and peptide biosynthesis. A total of seven DE-lncRNA target genes involved in muscle maintenance, embryonic skeletal joint morphogenesis, post-embryonic development and organ growth were identified in the E11 vs. E16, and E11 vs. D1 comparisons, including well-known genes that affect muscle growth, such as dystrophin (*DMD*) and delta like non-canonical notch ligand 1 (*DLK1*).

KEGG pathway analysis of DE-lncRNAs target genes was done to identify pathways that were enriched in DE-lncRNA target genes. For the E11 vs. E16, E11 vs. D1, and E16 vs. D1 comparisons, 18 (Table S13), 17 (Table S14), and one (Table S15) enriched pathways (*q* < 0.001) were detected, respectively. For the E11 vs. E16 comparison, 11 DE-lncRNAs target genes played a role in the most significantly enriched pathways, including fatty acid metabolism, valine, leucine and isoleucine degradation, fatty acid elongation in mitochondria, and benzoate degradation via CoA ligation. In contrast, for the E11 vs. D1 comparison, the mitogen-activated protein kinase (MAPK) signaling pathway, mismatch repair, beta-Alanine metabolism, and chondroitin sulfate biosynthesis were identified as the most significantly enriched pathways, with 13 DE-lncRNAs target genes being associated with these pathways.

### Verification of gene expression profiles using qRT-PCR

**Figure 8:**
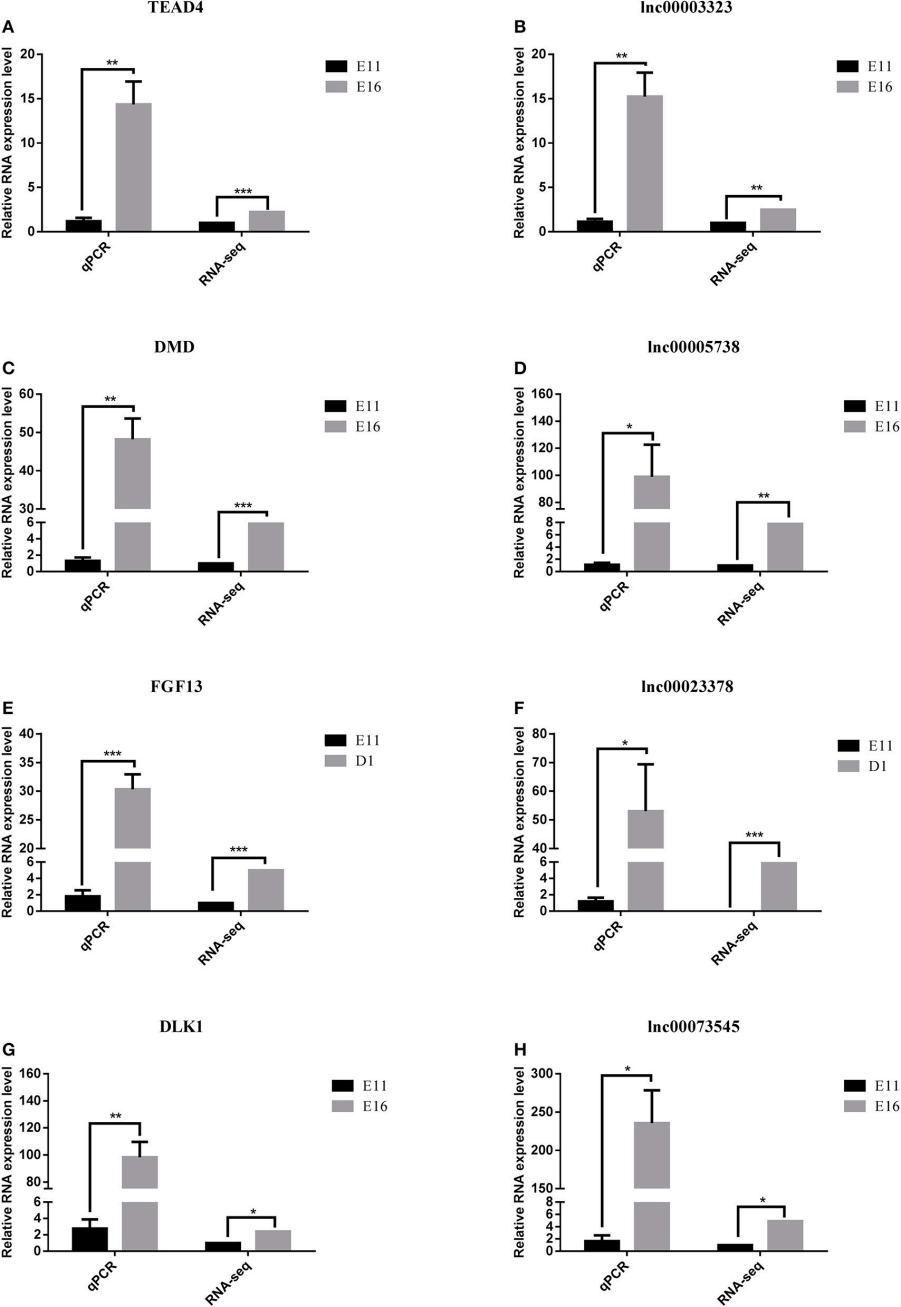
**Quantitative real-time PCR validation of differentially expressed lncRNAs and their corresponding target genes**. **(A)** The expression of TEAD4 was down-regulated in the E11 group compared with that in the E16 group. **(B)** The expression of lnc00003323 was down-regulated in the E11 group compared with that in the E16 group. **(C)** The expression of DMD was down-regulated in the E11 group compared with that in the E16 group. **(D)** The expression of lnc00005738 was down-regulated in the E11 group compared with that in the E16 group. **(E)** The expression of FGF13 was down-regulated in the E11 group compared with that in the D1 group. **(F)** The expression of lnc00023378 was down-regulated in the E11 group compared with that in the D1 group. **(G)** The expression of DLK1 was down-regulated in the E11 group compared with that in the E16 group. **(H)** The expression of lnc00073545 was down-regulated in the E11 group compared with that in the E16 group. RNA expression levels were determined by RT-qPCR analysis. Data are shown as means ± S.E.M. of at least three biological replicates. Asterisks denote statistically significant differences. ^*^, ^**^, and ^***^ indicate *P* < 0.05, *P* < 0.01, and *P* < 0.001, respectively.

To confirm the accuracy and reproducibility of the DE-lncRNA and DEG expression levels obtained from RNA-seq, four well-known DE-lncRNA target genes that affect muscle development, including the TEA domain transcription factor 4 (*TEAD4*) (Jacquemin et al., 1996), *DMD* (Ghahramani et al., 2010), fibroblast growth factor 13 (*FGF13*) (Itoh and Ornitz, 2008), and *DLK1* (Andersen et al., 2013), and their corresponding lncRNA regulators, lnc00003323, lnc00005738, lnc00023378, and lnc00073545, were selected for real-time qRT-PCR validation. The qRT-PCR primers for these lncRNAs and genes are shown in Table 1. All four lncRNAs and their target genes had similar expression patterns in comparison to the RNA-seq data (Figure 8), indicating the reliability of our RNA-seq data.

## Discussion

Over the past few years, many studies have demonstrated pervasive transcription across 70–90% of mammalian genomes (Wang and Chang, 2011; Derrien et al., 2012). Surprisingly, <2% of the genome encodes protein-coding genes, indicating that non-coding RNAs comprise the majority of the transcriptome. Based on their length, non-coding transcripts can generally be divided into two major classes. Small non-coding RNAs have been relatively well-characterized, and include piRNAs, small interfering RNAs (siRNAs), microRNAs (miRNAs), and some bacterial regulatory RNAs (Fatica and Bozzoni, 2014). In contrast, lncRNAs can vary in size from 200 bp to 100 kb, and have been implicated in imprinting control (Lee and Bartolomei, 2013), cell differentiation and development (Dong et al., 2014), and skeletal muscle growth (Kallen et al., 2013). However, the function of lncRNAs in chicken muscle development remains unclear. In the present study, Illumina high-throughput sequencing was performed to provide an extensive lncRNA and gene expression profile in the previously unexamined chicken leg skeletal muscle at stages E11, E16, and D1. Multiple comparisons of lncRNA and gene transcript levels allowed us to identify 129, 132, and 45 lncRNAs, and 1798, 3072, and 1211 genes that were differentially expressed in the E11 vs. E16, E11 vs. D1, and E16 vs. D1 comparisons, respectively. Li et al. have been reported in their study the first systematic identification of lncRNAs during the development of the chicken pectoralis muscle (Li T. et al., 2012). Using RNA-seq to sample the transcriptome, they successfully identified 281 new intergenic lncRNAs in the chicken genome. However, they did not identify the targets of lncRNAs or clarify their potential functions.

LncRNAs exert their effects by targeting protein-coding genes. There is accumulating evidence to suggest that lncRNAs are important factors that control gene expression through both *cis* and *trans*-acting regulatory mechanisms (Han et al., 2012). To gain insight into how interactions between lncRNAs and their target genes regulate muscle development, interaction networks composed of DE-lncRNAs and their predicted *cis*- and *trans*-acting targets were constructed. The information about the direction (sense or antisense) of lncRNA transcripts are presented in the Tables S1–S3, S7–S9. There is not a correlation between sense *cis*-acting lncRNA and gene activation or antisense *cis*-acting lncRNA and gene silencing based on data presented in Tables S7–S9. 54.03% of *cis*-acting lncRNAs is located in the upstream of their corresponding target genes, while 45.48% of *cis*-acting lncRNAs is located in the downstream of their corresponding target genes, and only 0.49% of *cis*-acting lncRNAs is located in their target genes body. Using IPA system, the function of lncRNA target genes were identified. Among three contrasts (E11 vs. E16, E11 vs. D1, E16 vs. D1), the lncRNA target genes of E11 vs. E16 comparisons were independently enriched in “cancer,” “organismal injury and abnormalities,” “cell cycle,” and “connective tissue development and function”; whereas the lncRNA target genes of E11 vs. D1 comparisons were independently enriched in “cell morphology,” “organ morphology,” “embryonic development,” “organismal development,” and “tissue development”; and the lncRNA target genes of E16 vs. D1 comparisons were independently enriched in “nervous system development and function” and “ophthalmic disease” (Table 3).

**Table 3:** **Summary of the most highly represented networks generated by IPA analysis**.

**Data**	**ID**	**Molecules in networks**	**Score**	**Focus gene**	**Top functions**
E11 vs. E16	1	**ACADL**; **ANO6**; **CAP2**; **COLEC11**;, Cg; Cyclin A; **DLK1**; **EGR1**; **EPCAM**; **ERBB4**; ERK1/2; **FGL2**; FSH; **GPSM1**; **HSD3B2**; Igm; Immunoglobulin; Lh; **MTUS1**; Mitochondrial complex 1; **NDUFA8**; **NELL2**; **NOD1**; Nos; Nr1h; **PITX1**; **PLIN1**; **PTPN21**; **PTX3**; **RGN**; **SHOX**; **SOD2**; **SOX6**; TCF; TSH	45	23	Cancer, organismal injury and abnormalities, cell cycle
	2	**ACAT2**; **ANKRD44**; Akt; **CPS1**; Ck2; **DAB1**; **DECR1**; **DUSP8**; **ECHS1**; **EPHA4**; **EPHX2**; **FABP7**; **G6PC2**; **HADHA**; **HADHB**; **IL16**; Jnk; **LAMA4**; LDL; **MTMR7**; **MYL3**; **PBK**; PDGF BB; PLC gamma; **PTN**; STAT5a/b; **STRBP**; **TEAD4**; **TSC22D1**; acetyl-CoA C-acetyltransferase; enoyl-CoA hydratase; hemoglobin; mediator; p85 (pik3r); phosphatase	45	22	Developmental disorder, hereditary disorder, metabolic disease
	3	26s Proteasome; ADRB, Actin; Alpha catenin; **CALB1**; CD3; **CDH8**; Creb; **DHCR24**; **DMD**; **EGFL6**; **EGLN3**; **FHDC1**; **FREM2**; Histone h3; Hsp90; Insulin; **MLF1**; **MYL1**; NFkB (complex); **NSDHL**; **PPP2R2B**; RNA polymerase II; **ROBO1**; **SLC20A2**; **STOM**; **SVIL**; Sos; **TIPARP**; **TNNT3**; **TSPAN9**; Ubiquitin; **WNT7B**; caspase; estrogen receptor	39	20	Cancer, organismal injury and abnormalities, reproductive system disease
	4	ABCA6; **ADAMTS18**; AMMECR1L; **ASB12**; CCND1; CDK1; CHUK; **CUTA**; **FAT4**; **FGL1**; **HOXD4**; HSF1; IL10; IL1A; **LECT2**; LZTS1; **MFAP5**; **MRPL18**; MYH10; **RHCG**; RUNX1; SEC16B; **SFXN5**; **SLC13A3**; SLC24A3; **SMAD9**; SMOC2; SPRR2B; SRPK2; STON1; **THNSL1**; TMPRSS11A; **ZFHX4**; beta-estradiol; cholesterol	27	15	Cellular development, cellular growth and proliferation, connective tissue development and function
E11 vs. D1	5	Actin; Alpha catenin; **CASP3**; **CDH4**; **CSRNP3**; **DCLK1**; **DMD**; ERK; F Actin; **GSN**; **LBR**; **MKL2**; **MYL3**; Mlc; **OSBP2**; P glycoprotein; **PAIP2**; PARP; **PARP1**; **PLEK2**; Pro-inflammatory Cytokine; **ROBO1**; SAA; **SCLT1**; **SH3PXD2A**; **SLC20A2**; **SLC38A2**; **STK39**; **STOM**; **SVIL**; **TNNT3**; Tnf (family); Troponin t; calpain; caspase	36	22	Cell morphology, organ morphology, reproductive system development and function
	6	**ASAH1**; **EPHA4**; **EPHB6**; ERK1/2; Eph Receptor; **FANCI**; **FGF13**; **FGFR1**; Integrin; JINK1/2; **LAMA4**; **MAP4K4**; **MTUS1**; N-cor; **NELL2**; Nfatc; Nr1h; **PAX3**; PEPCK; PLC gamma; **PLIN1**; **PPARGC1A**; **PTN**; Proinsulin; Rar; Rxr; **STRBP**; T3-TR-RXR; **TPT1**; **TSC22D1**; Wnt; **XYLT1**; **ZRANB1**; creatine kinase; histone deacetylase	33	19	Embryonic development, organismal development, tissue development
E16 vs. D1	7	**ACSL1**; ANGPTL1; Akt; Ap1; **CD82**; **CNR1**; **DKK3**; ERK; GPR135; IL12 (complex); IL12 (family); **IL15**; **IL18R1**; Igm; Immunoglobulin; Insulin; Jnk; MARVELD3; MUC8; Mapk; NFkB (complex); NMUR1; P38 MAPK; **PDK1**; PROKR1; **PTTG1**; SLC22A17; **SLC38A2**; **SLC4A7**; TAS1R2; TLR3/4; **TPD52L1**; TRAF1-TRAF2-TRAF3; ZNF675; ethylene glycol	27	11	Nervous system development and function, cellular development, cellular growth and proliferation
	8	ALKBH6; ARL17A/ARL17B; CCND1; CMC2; COQ10B; **COQ7**; **COTL1**; CPED1; CYP3A43; Ca2+; EXO5; **FREM2**; FUK; FUT10; GRIP1; GUF1; HHLA2; HNF1A; HNF1α dimer; HNF4A; **KCNIP1**; **NDUFA8**; **NDUFS3**; PAFAH2; PPCS; PRIMPOL; **RNF150**; RPA2; **SLC38A4**; **THNSL1**; TNF; UBE2D3; UGT2B11; ZNF502; mead acid	23	9	Developmental disorder, hereditary disorder, ophthalmic disease

*Gene symbols written in **bold** font indicate that the respective genes are DE-lncRNA target genes*.

Based on the lncRNA-gene interaction networks, *TEAD4* is predicted to be a target of lnc00003323. Moreover, the *TEAD4* gene plays an important role in the gene-gene network that is involved in developmental disorders and metabolic disease (Figure 7B). To our knowledge, prior to this analysis, no reports existed concerning the association between *TEAD4* and lncRNA. TEAD4 is a transcription factor that has a conserved TEA/ATTS DNA-binding domain, which recognizes the MCAT element in the promoters of muscle-specific genes (Benhaddou et al., 2012). In mouse embryos, *TEAD4* is specifically expressed in developing skeletal muscle tissue (Jacquemin et al., 1996). A previous study demonstrated that *TEAD4* is a direct target gene of the MYOD1 and MYOG transcription factors in the mouse C2C12 cell line (Jacquemin et al., 1996). In mouse 2-cell embryos, ablation of the mouse *TEAD4* gene leads to lack of trophectoderm specification and early preimplantation lethality (Benhaddou et al., 2012). Moreover, *TEAD4* is an important regulator of proliferation and organ size that plays a role in the Hippo signaling pathway of both *Drosophila* and mammalian cells (Zhao et al., 2010). The aforementioned studies indicate that *TEAD4* in particular, is an important regulator of muscle development. In the present study, *TEAD4* was expressed at higher levels at stage E16 than at stage E11, in skeletal muscles of the leg (Figure 8A). The predicted regulatory lncRNA, lnc00003323, could control the expression of *TEAD4* via *cis*-acting mechanisms, and is expressed at higher levels in skeletal muscle at stage E16 than at stage E11 (Figure 8B). Authors of a previous study have suggested that lncRNAs could activate the transcription of nearby genes in *cis* by recruiting remodeling factors to local chromatin (Luo et al., 2015). The positive relationship between lnc00003323 and *TEAD4* observed in the present study indicates that lnc00003323 targets *TEAD4* via *cis*-acting mechanisms to regulate skeletal muscle development in the chicken.

In the E11 vs. E16 comparison lncRNA-gene network (Figure 4), the *DMD* gene is the putative *cis*-target of lnc00005738. In addition, *DMD* plays an important role in gene-gene networks that are involved in organismal injury and abnormalities, cell morphology and organ morphology (Figures 7C,E). The *DMD* gene is the largest gene described in human beings that encodes a rod-like cytoskeletal protein that is found at the inner surface of muscle fibers in skeletal and cardiac muscles (Muntoni et al., 2003). The encoded protein, dystrophin, is required for proper development and organization of myofibers, as contractile units in striated muscles (Ghahramani et al., 2010).

In the E11 vs. D1 comparison lncRNA-gene network (Figure 5), *FGF13* is a predicted *cis*-target of lnc00023378 that is a part of the gene-gene interaction network that is related to embryonic development, organismal development, and tissue development (Figure 7F). *FGF13* is a member of the fibroblast growth factor (FGF) family. Members of this family possess broad mitogenic and cell survival activities, and are involved in a variety of biological processes, including embryonic development, cell growth, morphogenesis, tissue repair, tumor growth, and invasion (Itoh and Ornitz, 2008). A recent study demonstrated that *FGF13* regulates proliferation and differentiation of skeletal muscle by down-regulating *Spry1* (Lu et al., 2015). *DLK1*, which encodes a transmembrane protein, is the putative *cis*-target of lnc00073545 (Figure 4). *DLK1* is an imprinted gene, the increased expression of which is associated with muscle hypertrophy in animal models. As with many imprinted genes, *DLK1* plays an important role in mammalian development, and its expression level exerts remarkable effects on cell proliferation and differentiation (Waddell et al., 2010). Previous research has shown that *DLK1* is a crucial regulator of the myogenic program in skeletal muscle. During embryogenesis, *DLK1* knockout mice display developmental delays in gaining muscle mass and function, owing to inhibition of the myogenic program (Andersen et al., 2013). In the present study, lnc00073545 and *DLK1* were expressed at higher levels in skeletal muscle of the leg at stage E16, as compared to stage E11 (Figures 8G,H). The positive correlation between lnc00073545 and *DLK1* expression patterns suggest that lnc00073545 might play an important role in skeletal muscle development in the chicken via its *cis*-regulation of *DLK1*.

In summary, we have identified a set of lncRNAs and genes that are differentially expressed at three different stages of skeletal muscle development in the chicken. The functions of, and biological processes associated with lncRNAs in skeletal muscle development were determined, based on IPA analysis and GO and KEGG pathway annotations of lncRNA target genes. The results of the *cis*- and *trans*-acting lncRNA target gene analyses and the lncRNA-gene interaction networks provide information that could be utilized in further studies of lncRNA function in skeletal muscle development in the chicken.

## Author contributions

ZL: performed research, analyzed data, and wrote the paper. HO: analyzed data and was involved in the study design. MZ, BC, PH: analyzed data. BA: reviewed the manuscript. QN conceived the study, and was involved in its design and coordination. XZ was involved in the design of the study.

## Funding

This research was supported by the Program for New Century Excellent Talents in University (NCET-13-0803) and the Foundation for High-level Talents in Higher Education of Guangdong, China.

### Conflict of interest statement

The authors declare that the research was conducted in the absence of any commercial or financial relationships that could be construed as a potential conflict of interest.

## Abbreviations

CNCI: Coding-Non-Coding Index
CPC: Coding Potential Calculator
DEG: differentially expressed genes
FGF: fibroblast growth factor
GO: gene ontology
IPA: Ingenuity Pathway Analysis
IPKB: Ingenuity Pathways Knowledge Base
KEGG: Kyoto Encyclopedia of Genes and Genomes
PhyloCSF: Phylogenetic codon substitution frequency
*DMD*: dystrophin gene (gene that causes Duchenne muscular dystrophy)
LncRNA: long non-coding RNA.
