# Supplementary material for: Integrated Analysis of Long Non-coding RNAs (LncRNAs) and mRNA Expression Profiles Reveals the Potential Role of LncRNAs in Skeletal Muscle Development of the Chicken
Source: Front Physiol. 2017 Jan 9;7:687. doi: 10.3389/fphys.2016.00687 (PMC5220077; doi:10.3389/fphys.2016.00687)
Supplement: Supplementary file 16 [file Image1.PDF]

Figure S1 Histogram of gene ontology (GO) classification. GO analysis of **(A)** E11 vs. E16 **(B)** E11 vs. D1 and. **(C)** E16 vs. D1 DE-lncRNAs target genes. Blue bars: biological process; Red bars: cellular component; Yellow bars: molecular function.

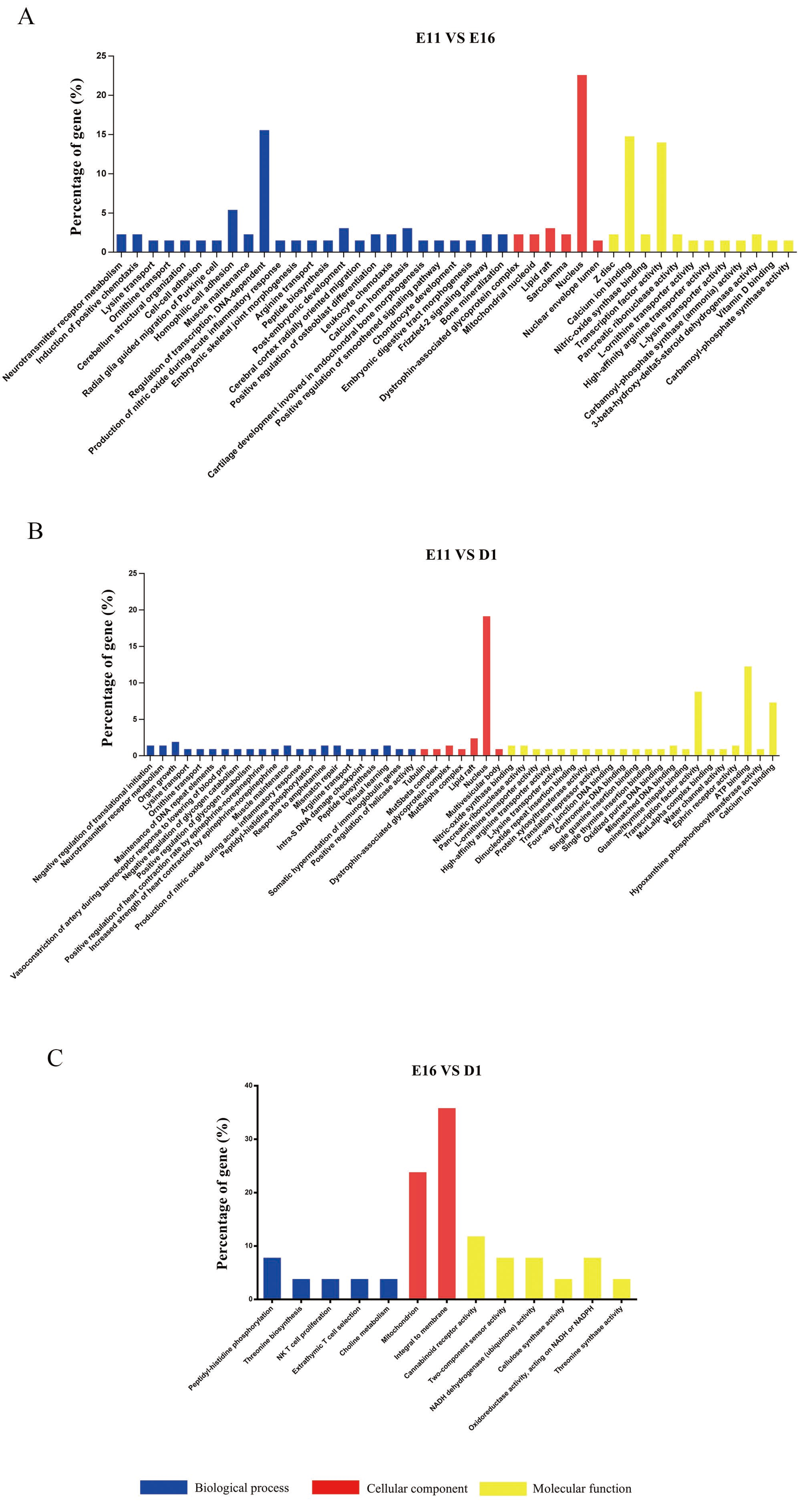
A
E11 VS E16
Percentage of gene (%)
0
5
10
15
20
25
Neurotransmitter receptor metabolism
Induction of positive chemotaxis
Lysine transport
Ornithine transport
Cerebellum structural organization
Cell-cell adhesion
Radial glia guided migration of Purkinje cell
Homophilic cell adhesion
Muscle maintenance
Regulation of transcription, DNA-dependent
Production of nitric oxide during acute inflammatory response
Embryonic skeletal joint morphogenesis
Arginine transport
Peptide biosynthesis
Post-embryonic development
Cerebral cortex radially oriented migration
Positive regulation of osteoblast differentiation
Leukocyte chemotaxis
Calcium ion homeostasis
Cartilage development involved in endochondral bone morphogenesis
Positive regulation of smoothened signaling pathway
Chondrocyte development
Embryonic digestive tract morphogenesis
Frizzled-2 signaling pathway
Bone mineralization
Dystrophin-associated glycoprotein complex
Mitochondrial nucleoid
Lipid raft
Sarcolemma
Nucleus
Nuclear envelope lumen
Z disc
Calcium ion binding
Nitric-oxide synthase binding
Transcription factor activity
Pancreatic ribonuclease activity
L-ornithine transporter activity
High-affinity arginine transporter activity
L-lysine transporter activity
Carbamoyl-phosphate synthase (ammonia) activity
3-beta-hydroxy-delta5-steroid dehydrogenase activity
Vitamin D binding
Carbamoyl-phosphate synthase activity
B
E11 VS D1
Percentage of gene (%)
0
5
10
15
20
25
Negative regulation of translational initiation
Neurotransmitter receptor metabolism
Organ growth
Lysine transport
Ornithine transport
Maintenance of DNA repeat elements
Vasoconstriction of artery during baroreceptor response to lowering of blood pre
Negative regulation of glycogen catabolism
Positive regulation of glycogen catabolism
Positive regulation of heart contraction rate by epinephrine-norepinephrine
Increased strength of heart contraction by epinephrine-norepinephrine
Muscle maintenance
Production of nitric oxide during acute inflammatory response
Peptidyl-histidine phosphorylation
Response to amphetamine
Mismatch repair
Arginine transport
Intra-S DNA damage checkpoint
Peptide biosynthesis
Visual learning
Somatic hypermutation of immunoglobulin genes
Positive regulation of helicase activity
Tubulin
MutSbeta complex
Dystrophin-associated glycoprotein complex
MutSalpha complex
Lipid raft
Nucleus
Multivesicular body
Nitric-oxide synthase binding
Pancreatic ribonuclease activity
L-ornithine transporter activity
High-affinity arginine transporter activity
L-lysine transporter activity
Dinucleotide repeat insertion binding
Protein xylosyltransferase activity
Translation repressor activity
Four-way junction DNA binding
Centromeric DNA binding
Single guanine insertion binding
Single thymine insertion binding
Oxidized purine DNA binding
Mismatched DNA binding
Guanine/thymine mispair binding
Transcription factor activity
MutLalpha complex binding
Water channel activity
Ephrin receptor activity
ATP binding
Hypoxanthine phosphoribosyltransferase activity
Calcium ion binding
C
E16 VS D1
Percentage of gene (%)
0
10
20
30
40
Peptidyl-histidine phosphorylation
Threonine biosynthesis
NK T cell proliferation
Extrathymic T cell selection
Choline metabolism
Mitochondrion
Integral to membrane
Cannabinoid receptor activity
Two-component sensor activity
NADH dehydrogenase (ubiquinone) activity
Cellulose synthase activity
Oxidoreductase activity, acting on NADH or NADPH
Threonine synthase activity
Biological process
Cellular component
Molecular function
